# Isoflavones and probiotics effect on bone calcium and bone cells in rats

**DOI:** 10.1016/j.heliyon.2023.e16801

**Published:** 2023-05-29

**Authors:** Iskandar Azmy Harahap, Maciej Kuligowski, Marcin Schmidt, Paweł Kurzawa, Ewa Pruszyńska-Oszmałek, Maciej Sassek, Joanna Suliburska

**Affiliations:** a Department of Human Nutrition and Dietetics, Faculty of Food Science and Nutrition, Poznań University of Life Sciences, Poznań, Poland; b Department of Food Technology of Plant Origin, Faculty of Food Science and Nutrition, Poznań University of Life Sciences, Poznań, Poland; c Department of Biotechnology and Food Microbiology, Faculty of Food Science and Nutrition, Poznań University of Life Sciences, Poznań, Poland; d Department of Clinical Pathology, Poznań University of Medical Sciences, Poznań, Poland; e Department of Animal Physiology, Biochemistry and Biostructure, Faculty of Veterinary Medicine and Animal Science, Poznań University of Life Sciences, Poznań, Poland

**Keywords:** Isoflavones, *Lactobacillus acidophilus*, Calcium status, Bone cells, Rats

## Abstract

Isoflavones and probiotics have shown the therapeutic potential to alter calcium absorption and bone cell metabolism. This study sought to ascertain the effect of isoflavones and probiotics on calcium status and bone health in healthy female rats. Forty-eight adult female Wistar rats were grouped and fed: a standard diet (control); and standard diets with tempeh; soy; daidzein and genistein; *Lactobacillus acidophilus*; and a combination of daidzein, genistein, and *L. acidophilus*. The biochemical serum parameters, such as alanine transaminase, aspartate transaminase, glucose, and triacylglycerol concentrations, were measured, and calcium contents in tissues were determined. After staining the bone with hematoxylin and eosin, the number of osteoblasts, osteocytes, and the percentage of bone marrow adipocytes were counted. Compared with the control group, the soy group showed a significantly lower triacylglycerol concentration. The L. *acidophilus* group considerably increased the calcium content in the femoral bone. The daidzein and genistein, *L. acidophilus*, and a combination of daidzein, genistein, and *L. acidophilus* groups showed significantly lower calcium contents in the heart and kidneys. The daidzein and genistein group significantly enhanced the number of osteoblasts and osteocytes. A substantial inverse correlation was observed between calcium contents in kidneys and osteoblasts. In conclusion, the combination of daidzein, genistein, and *L. acidophilus* may improve bone calcium concentrations and bone cells. However, no synergistic effect between isoflavones and probiotics was detected in this study.

## 1 Introduction

Calcium ions (Ca^2+^) are vital for living organisms for muscle contraction, pancreatic insulin, and glucagon production in blood glucose (GLU) concentrations [1]. Calcium is also a structurally important component of bones. Therefore, to prevent osteoporosis and fractures by maintaining healthy bones throughout life, a diet rich in calcium and regular weight-bearing physical exercise is necessary [2].

About 99% of the body's calcium supply is stored in the bones and teeth, making bones the reservoirs for calcium homeostasis [3]. Extracellular calcium regulates bone remodeling by directly regulating parathyroid cells via the calcium-sensing receptor (CaSR). It promotes osteoblast chemotaxis and proliferation in humans, and the CaSR may play a role in osteoblast differentiation [4]. Biological processes, such as bone differentiation, bone resorption, and gene transcription, rely on Ca^2+^ signals in osteoclasts [5]. Bone growth abnormalities and osteoporosis are caused by dysfunction in the effector cells responsible for bone creation and resorption, osteoblasts, and osteoclasts. Osteocytes—cells present deep inside the bone matrix—are increasingly understood to be the key local orchestrators of many bone processes. In particular, they secrete soluble signaling molecules that control Ca^2+^ and phosphate transport in the bone matrix, in addition to bone production and resorption [6]. Several factors influence the risk of developing osteoporosis, such as genetics, endocrine function, physical activities, and diet, with calcium status being an important contributor [7]. An adequate and appropriate amount of calcium is needed for bone health, positively influencing calcium absorption and optimizing calcium bioavailability from food products.

Calcium bioavailability is a crucial factor in maintaining bone health. Calcium bioavailability is related to calcium status and metabolism, and consequently to bone metabolism. Calcium bioavailability is affected by various factors, including internal and external variables. Some factors can hinder calcium absorption, while others can enhance it. Certain drugs, food components, and dietary changes can lower calcium levels and contribute to osteoporosis. Therefore, it is crucial to maintain a balanced diet that promotes calcium bioavailability to improve bone density and reduce the risk of osteoporosis [8]. For example, research has shown that calcium complexes derived from soybean protein can positively impact bone mass in rapidly growing rats [9]. Additionally, probiotics have increased calcium absorption and bone weight by 35% compared to the control group [10].

Probiotics and isoflavones are promising therapeutic nutrients for regulating calcium absorption and bone cell metabolism. Probiotics can modulate calcium uptake via paracellular or transcellular pathways. Isoflavones are involved in calcium homeostasis by releasing skeletal calcium into the bloodstream. Bone health depends on the gut microbiota and the immune system, both of which are improved by the presence of *Lactobacillus* and *Bifidobacteria*. Bone mineral density can be increased by consuming isoflavones and their metabolites [11]. The gut microbiome consists of billions of microorganisms that play a crucial role in various biological processes, including inflammation control, immunology, and homeostasis, by interacting with the host. An important factor that indicates the risk of developing osteoporosis is the ratio of Firmicutes to Bacteroidetes bacteria in the gut. These bacteria are essential for the differentiation of osteoclasts in the intestine. Additionally, Firmicutes and Bacteroidetes regulate the de novo synthesis of glutathione and inhibit the generation of reactive oxygen species by controlling the activity of the glutamate-cysteine ligase catalytic subunit and the cAMP response element-binding pathway [12].

Our previous studies demonstrated that the oral supplementation of a combination of daidzein, genistein, and *L. acidophilus* could significantly improve the fecal *Lactobacillus* spp. Status in healthy female rats [13]. *L. acidophilus* has osteoprotective properties that can promote bone health. By suppressing osteoclastogenic Th17 cells and boosting antiosteoclastogenic Treg cells, *L. acidophilus* alters the Treg–Th17 cell balance in ovariectomized mice. Antiosteoclastogenic factors (IL-10 and IFN-γ) were upregulated, whereas osteoclastogenic factors (IL-6, IL-17, TNF-α, and RANKL) were downregulated after *L. acidophilus* administration [14].

In the meantime, daidzein is widely available in soy and soy products such as fermented soy, most notably tempeh, in which the isoflavone content is higher than in unfermented soy [15]. Intestinal microflora converts daidzein to equol [16], and equol prevents bone degeneration in ovariectomized mice [17]. The bioactive peptides present in tempeh flour have been shown to improve nutritional status biomarkers through improving insulin-like growth factor-1 [18]. However, Astawan et al. found that consuming tempeh and soybean flour as a source of protein did not significantly alter the body's ability to absorb calcium [19]. Moreover, Galán & Drago showed that the mineral bioaccessibility of soy products decreased with increasing soy protein concentration [20]. Thus, in response to the contradictory findings of earlier research, this study aimed to determine how eight weeks of oral supplementation of isoflavones and *L. acidophilus* affected calcium status, selected bone cells, and biochemical parameters in healthy female rats.

## 2 Material and methods

### 2.1 Experimental design

This study was conducted in accordance with the Act of January 15, 2015 (Journal of Laws of 2015, item 266), following the National Institute of Health's Guide for the Care and Use of Laboratory Animals (NIH Publication No. 80–23, Revised 1978), as well as the European Communities Council Directive of November 24, 1986, and Polish law. Animal experiments were carried out according to the guidelines established by Poznań University of Life Sciences by adhering to the Animal Research: Reporting of In Vivo Experiments (ARRIVE) guidelines.

**Fig. 1:**
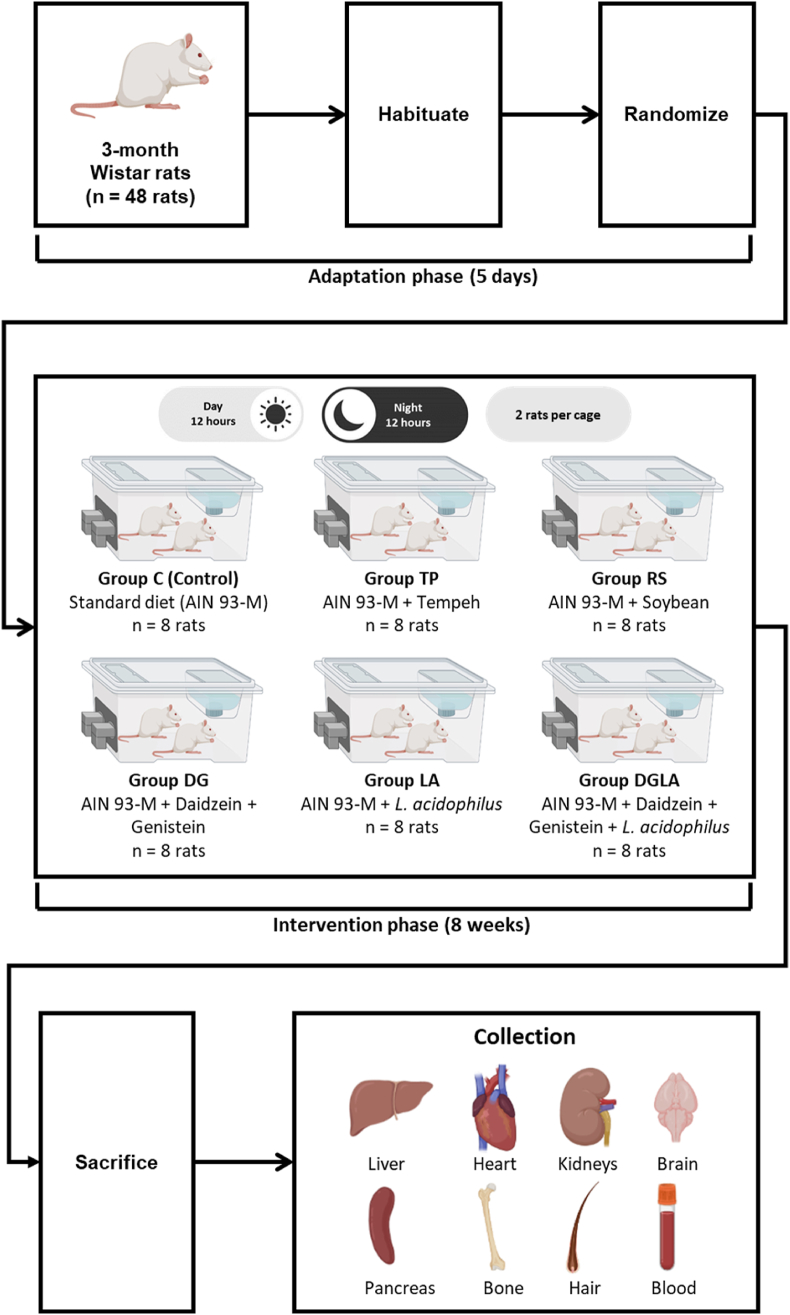
Scheme of adaptation, intervention, and organs collection in rats. C: control; TP: tempeh; RS: soy; DG: daidzein and genistein; LA: *Lactobacillus acidophilus*; DGLA: daidzein and genistein and *Lactobacillus acidophilus*.

The female Wistar rats were housed in a regulated and stable environment in the Animal Laboratory of Poznań University of Life Sciences, Poland. During the adaptation period and the experiment, the relative humidity of the room in which the rats were kept ranged from 55 to 65%. The temperature was constant at 21 ± 2 °C, and the rats were exposed to light and dark cycles lasting 12 h each. The rats were housed in pairs in metal-free, enamel-coated stainless steel cages throughout the adaptation and intervention phases. During the first 5 days, the rats became accustomed to the controlled conditions of the laboratory. A flowchart detailing the research process is presented in Fig. 1.

After the adaptation period, all rats were weighed on a calibrated scale. Then, they were randomized into six groups (eight rats each) based on their body weight. Research involving a minimum of eight rats has been shown to have adequate power to detect a statistically significant intervention effect. Moreover, ethical considerations prohibit using more rats in one study group [21].

The control (C) group was fed the AIN 93 M standard diet (Zoolab, Sdziszów, Poland), and the composition of the standard diet and other modified diets was determined as described in our previous paper [13]. The tempeh (TP) group received AIN 93 M with tempeh flour (250 g/kg of standard diet), whereas the soy group received AIN 93 M with soy *Glycine* max variety Augusta (RS) flour (250 g/kg of standard diet). The daidzein and genistein (DG) group was provided AIN 93 M supplemented with daidzein (10 mg/kg of standard diet) and genistein (100 mg/kg of standard diet) purchased from LC Laboratories (Woburn, MA, USA). The probiotics (LA) group was provided AIN 93 M supplemented with *Lactobacillus acidophilus* DSM079 (10^10^ CFU/day) obtained from the German Collection of Microorganisms and Cell Cultures, the Leibniz Institute DSMZ, Germany. The daidzein, genistein, and probiotics (DGLA) group received AIN 93 M with daidzein (10 mg/kg of standard diet), genistein (100 mg/kg of standard diet), and *L. acidophilus* DSM079 (10^10^ CFU/day). Tempeh (fermented soy with *Rhizopus oligosporus* NRRL 2710) and the probiotic (*Lactobacillus acidophilus* DSM079) used in this study were prepared following the method of Harahap et al. [13]. To standardize the content of daidzein and genistein in this study, the amount of these compounds was adjusted to the content present in 250 g of tempeh. In addition, the proximate nutritional composition of each diet and isoflavones content of soybean and tempeh were reported in our previous result [13].

All groups had free access to diet and distilled water. The rats were weighed weekly, and their food intake was recorded daily. After 8 weeks of intervention, the rats were fasted for 12 h. Then, their body mass was measured, and they were euthanized by decapitation.

### 2.2 Collecting blood and tissues

The rats were treated to a 12-h fast following an 8-week intervention. Following a measurement of their bodily mass, their heads were severed. Immediately upon decapitation, a section was carried out. The total blood sample was collected through cardiac puncture. Dissected tissues were cleaned in saline, weighed, and frozen at −80 °C. The tissues included the liver, heart, kidney, pancreas, femur, and brain. In addition, hair samples were shaved from the same locations on all rats (the interscapular region). The following biochemical parameters were measured in a commercial laboratory (Alab, Poznań, Poland): alanine transaminase (ALT), aspartate transaminase (AST), glucose (GLU), and triacylglycerol (TG) concentrations.

### 2.3 Determination of calcium contents in diets and organs

Calcium contents in diets were determined following the method of Suliburska et al. [22]. In brief, 2 g of each diet was weighed, ashed in a muffle furnace at 450 °C until complete mineralization, and dissolved in 1 N nitric acid (Suprapure, Merck). Then, calcium concentrations were determined using flame atomic absorption spectrometry (AAS-3, Carl Zeiss, Jena, Germany).

Calcium contents in the liver, heart, kidney, pancreas, femur, brain, and hair were measured after digestion in 65% (w/w) spectra pure HNO3 (Merck, Kenilworth, NJ, USA) using a Microwave Digestion system (Speedwave Xpert, Berghof, Eningen, Germany), following the procedure of Suliburska et al. [23]. After digestion and subsequent dilution with deionized water, calcium contents in the mineral solutions were measured using flame atomic absorption spectrometry (AAS-3, Carl Zeiss, Jena, Germany).

Calcium contents in diets and organs were measured at a wavelength of 422.7 nm. The reliability of this method was confirmed using certified reference materials. A certified reference material of soybean flour INCT-SBF-4 (Institute of Nuclear Chemistry and Technology, Poland) was used to determine calcium contents in diets with a 92% accuracy. In addition, a certified reference material of bovine liver NIST-1577C (Sigma-Aldrich, Saint Louis, MO, USA) was used to determine tissue calcium contents with a 91% accuracy.

### 2.4 Hematoxylin and eosin (H&E) staining bone

Femoral bones used as specimens were fixed in 10% buffered formalin after surgery for 24 h. The samples were then submerged for 3 h in a decalcifying solution (Osteodec bone marrow biopsy decalcifying solution). Then, the bone specimens were treated and embedded in paraffin blocks individually according to the standard operating procedure. Three 2-μm slices were cut from the paraffin blocks and stained with H&E. There were two femoral bone slices with their bone marrow content on each slide. Two researchers independently examined the number of osteoblasts, the number of osteocytes, and the percentage of bone marrow adipocytes of each sample in a high-power field using a light microscope (Leica, Allendale, NJ, USA).

### 2.5 Statistical analysis

Tukey's post hoc honestly significant difference test was used to determine statistical significance after conducting an analysis of variance (ANOVA). The Shapiro–Wilk test was used to check the normality of variable distributions. All differences were considered statistically significant at the 5% probability level. SPSS 22 for Windows was used for statistical analysis. The calcium content in diets was measured thrice, and all data were expressed as mean ± standard deviations. Spearman's correlation was used to investigate the association between calcium contents in tissues, blood parameters, and femoral bone cells.

## 3 Results

### 3.1 Food consumption characteristics

In all diets, there were no significant differences in the calcium content (Table 1). The intake of diet was the highest in the daidzein and genistein group and the lowest in the soybean group. Therefore, calcium consumption was also highest in the daidzein and genistein group and the lowest in the soy group. Meanwhile, calcium intake was considerably higher in the daidzein and genistein, *L. acidophilus,* and the combination of daidzein, genistein, and *L. acidophilus* groups and significantly lower in the tempeh and soy groups compared with the control group.

**Table 1:** Feeding intake and calcium intake.

Parameters	Unit	Number of samples per group	Group					
			C	TP	RS	DG	LA	DGLA
Feeding intake	g/day	8	18.89 ± 2.14^c^	18.20 ± 2.06^b^	17.30 ± 2.65^a^	22.48 ± 2.39^e^	21.85 ± 2.25^d^	21.49 ± 1.98^d^
Calcium content	mg/g dry mass	3*	4.95 ± 0.05	4.94 ± 0.04	4.93 ± 0.06	5.00 ± 0.09	4.91 ± 0.02	4.96 ± 0.01
Calcium intake	mg/day	8	93.52 ± 10.62^c^	89.76 ± 10.18^b^	85.18 ± 13.09^a^	112.22 ± 12.03^e^	107.16 ± 11.15^d^	106.54 ± 9.84^d^

C: control; TP: tempeh; RS: soy; DG: daidzein and genistein; LA: *Lactobacillus acidophilus*; DGLA: daidzein and genistein and *Lactobacillus acidophilus*. Results of ANOVA analysis followed by Tukey's post hoc honestly significant difference test showing significant differences between groups, denoted by different superscript letters (^a, b, c, d, e^) and presented as mean ± SD. *: Calcium concentration in diets was analyzed in triplicate.

### 3.2 Impact on body growth

Body mass and organ mass are presented in Table 2. After the 8-week intervention, the end-line body mass in the groups with modified diets did not differ significantly from that of the control group. The soy group showed a significantly reduced body mass compared with the daidzein and genistein, *L. acidophilus,* and the combination of daidzein, genistein, and *L. acidophilus* groups. In contrast to the control and *L. acidophilus* groups, the soy group showed a significantly higher relative weight of the kidneys. Furthermore, compared with other groups, the soy group showed a significantly higher relative weight of the pancreas. Compared with the tempeh, daidzein and genistein, *L. acidophilus*, and the combination of daidzein, genistein, and *L. acidophilus* groups, a significantly higher relative weight of the brain was observed in the soy group.

**Table 2:** Body mass and relative weight of tissues*.

Parameters	Unit	Number of samples per group	Group					
			C	TP	RS	DG	LA	DGLA
Endline body mass	g	8	284.63 ± 11.04^ab^	292.25 ± 22.81^ab^	263.88 ± 20.82^a^	309.88 ± 30.46^b^	312.38 ± 22.96^b^	307.00 ± 22.13^b^
Liver relative weight	%	8	2.45 ± 0.18	2.47 ± 0.19	2.63 ± 0.18	2.68 ± 0.19	2.59 ± 0.16	2.59 ± 0.21
Heart relative weight	%	8	0.31 ± 0.03	0.30 ± 0.02	0.30 ± 0.03	0.31 ± 0.03	0.29 ± 0.02	0.30 ± 0.02
Kidney relative weight	%	8	0.58 ± 0.05^a^	0.64 ± 0.04^ab^	0.65 ± 0.04^b^	0.60 ± 0.03^ab^	0.58 ± 0.06^a^	0.60 ± 0.05^ab^
Pancreas relative weight	%	8	0.32 ± 0.06^a^	0.35 ± 0.07^a^	0.61 ± 0.08^b^	0.32 ± 0.06^a^	0.30 ± 0.04^a^	0.31 ± 0.05^a^
Femurs relative weight	%	8	0.59 ± 0.03	0.61 ± 0.05	0.67 ± 0.04	0.53 ± 0.22	0.60 ± 0.05	0.59 ± 0.03
Brain relative weight	%	8	0.65 ± 0.03^ab^	0.62 ± 0.06^a^	0.71 ± 0.08^b^	0.59 ± 0.06^a^	0.59 ± 0.06^a^	0.60 ± 0.05^a^

C: control; TP: tempeh; RS: soy; DG: daidzein and genistein; LA: *Lactobacillus acidophilus*; DGLA: daidzein and genistein and *Lactobacillus acidophilus*. Results of ANOVA analysis followed by Tukey's post hoc honestly significant difference test showing significant differences between groups, denoted by different superscript letters (^a, b^) and presented as mean ± SD. *: Relative tissue weights of rats expressed as a percentage of body weight, and relative weight was calculated by dividing tissue weight by body mass and multiplying by 100%.

### 3.3 Impact on selected biochemical parameters

Serum concentrations of biochemical parameters, namely alanine transaminase (ALT), aspartate transaminase (AST), glucose (GLU), and triacylglycerol (TG), are presented in Table 3. Even though there were no discernible differences with the control group, the soy group showed a substantial increase in AST concentrations compared with the *L. acidophilus* group. The soy group showed significantly lower GLU concentrations than the combination of daidzein, genistein, and *L. acidophilus* group and significantly lower TG concentrations than the control, daidzein and genistein, and *L. acidophilus* groups.

**Table 3:** Biochemical parameters concentration in serum.

Parameters	Unit	Number of samples per group	Group					
			C	TP	RS	DG	LA	DGLA
ALT	U/l	8	35.87 ± 7.48	38.96 ± 11.65	43.75 ± 7.61	39.25 ± 9.32	33.00 ± 4.28	36.25 ± 5.87
AST	U/l	8	214.29 ± 58.38^ab^	266.88 ± 35.22^ab^	293.38 ± 66.25^b^	235.13 ± 83.64^ab^	197.00 ± 15.64^a^	212.75 ± 43.97^ab^
GLU	mg/dl	8	121.75 ± 11.26^ab^	116.00 ± 7.58^ab^	109.63 ± 8.31^a^	119.50 ± 5.28^ab^	118.75 ± 11.20^ab^	124.71 ± 7.39^b^
TG	mg/dl	8	71.53 ± 14.99^bc^	47.43 ± 9.93^ab^	42.43 ± 8.18^a^	80.86 ± 21.34^c^	69.25 ± 18.25^bc^	66.43 ± 16.26^abc^

C: control; TP: tempeh; RS: soy; DG: daidzein and genistein; LA: *Lactobacillus acidophilus*; DGLA: daidzein and genistein and *Lactobacillus acidophilus*. ALT: alanine transaminase; AST: aspartate transaminase; GLU: glucose; TG: triacylglycerol. Results of ANOVA analysis followed by Tukey's post hoc honestly significant difference test showing significant differences between groups, denoted by different superscript letters (^a, b^) and presented as mean ± SD.

### 3.4 Impact on calcium contents in tissues

Calcium contents in tissues after 8 weeks of intervention are presented in Table 4. All treatment groups showed considerably lower calcium contents in the heart than the control group. Compared with the control group, the daidzein and genistein, *L. acidophilus*, and the combination of daidzein, genistein, and *L. acidophilus* groups showed a noticeable decline in kidney calcium contents. In addition, pancreatic calcium contents were considerably increased in the soy group compared with other groups. The L. *acidophilus* group showed significantly higher calcium contents in the femur than the control and the combination of daidzein, genistein, and *L. acidophilus* groups.

**Table 4:** Calcium contents in tissues.

Parameters	Unit	Number of samples per group	Group					
			C	TP	RS	DG	LA	DGLA
Liver	μmol/g dry mass	8	1.34 ± 0.40	1.34 ± 0.13	1.71 ± 0.26	1.70 ± 0.12	1.74 ± 0.33	1.75 ± 0.28
Heart	μmol/g dry mass	8	1.87 ± 0.23^c^	1.02 ± 0.57^b^	0.45 ± 0.22^a^	0.45 ± 0.11^a^	0.27 ± 0.22^a^	0.28 ± 0.12^a^
Kidney	μmol/g dry mass	8	3.29 ± 0.39^b^	3.44 ± 0.56^b^	3.56 ± 1.29^b^	1.07 ± 0.27^a^	1.12 ± 0.18^a^	1.14 ± 0.31^a^
Pancreas	μmol/g dry mass	8	4.39 ± 0.60^a^	4.03 ± 0.74^a^	6.60 ± 1.16^b^	3.64 ± 0.92^a^	4.51 ± 0.66^a^	3.98 ± 0.78^a^
Femur	μmol/g dry mass	8	3.73 ± 0.27^a^	4.04 ± 0.35^ab^	4.01 ± 0.24^ab^	3.99 ± 0.60^ab^	4.58 ± 0.88^b^	3.78 ± 0.31^a^
Brain	μmol/g dry mass	8	1.68 ± 0.91	1.29 ± 0.89	1.06 ± 0.39	0.92 ± 0.29	1.38 ± 0.55	0.77 ± 0.24
Hair	μmol/g dry mass	8	8.74 ± 2.45	8.42 ± 2.66	9.57 ± 1.79	7.52 ± 1.49	9.02 ± 1.08	7.20 ± 1.33

C: control; TP: tempeh; RS: soy; DG: daidzein and genistein; LA: *Lactobacillus acidophilus*; DGLA: daidzein and genistein and *Lactobacillus acidophilus*. Results of ANOVA analysis followed by Tukey's post hoc honestly significant difference test showing significant differences between groups, denoted by different superscript letters (^a, b, c^) and presented as mean ± SD.

### 3.5 Impact on femoral bone cells

**Fig. 2:**
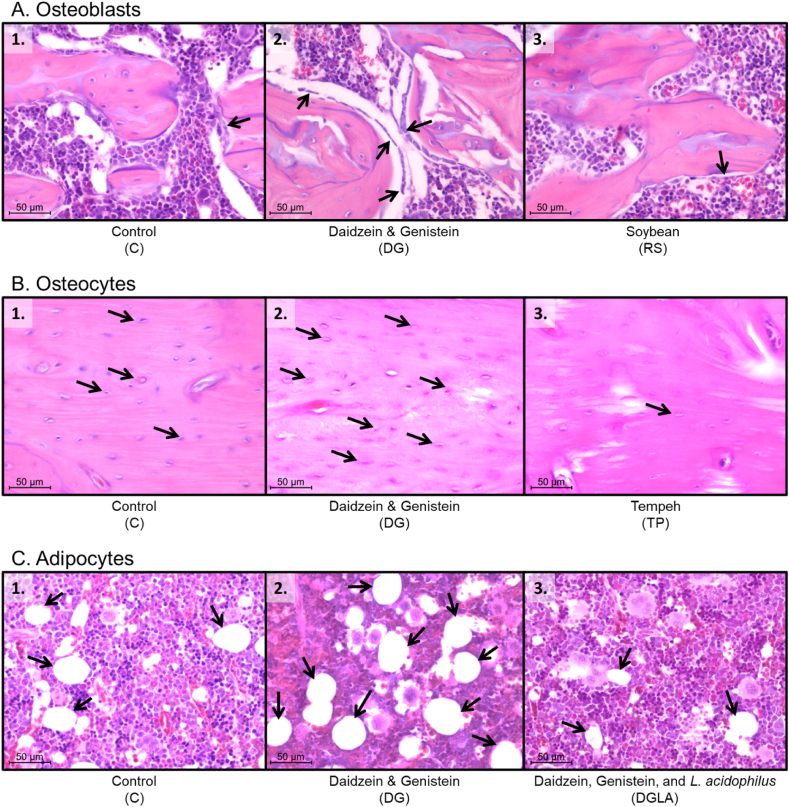
Comparison of osteoblasts (A), osteocytes (B), and adipocytes (C) in the femoral bone marrow. A.2. Represents the group with the most osteoblasts (Group DG; 400 × magnification), and A.3. Represents the group with the fewest osteoblasts (Group RS; 400 × magnification) compared with the A.1. Represents the control group (Group C; 400 × magnification); B.2. Represents the group with the most osteocytes (Group DG; 400 × magnification), and B.3. Represents the group with the fewest osteocytes (Group TP; 400 × magnification) compared with the B.1. Represents the control group (Group C; 400 × magnification); C.2. Represents the group with the greatest number of adipocytes (Group DG; 400 × magnification), and C.3. Represents the group with the fewest adipocytes (Group DGLA; 400 × magnification) compared with the C.1. Represents the control group (Group C; 400 × magnification).

Following the 8-week intervention research, the numbers of osteoblasts, osteocytes, and bone marrow adipocytes in the bone femur of healthy female rats are presented in Table 5, along with the findings of H&E staining (Fig. 2). The numbers of osteoblasts and osteocytes were markedly higher in the daidzein and genistein group compared with the control group.

**Table 5:** Number of osteoblasts, number of osteoclasts, and percentage of bone marrow femoral adipocytes in femoral bones.

Parameter	Number of samples per group	Group					
		C	TP	RS	DG	LA	DGLA
Osteoblasts	8	26.13 ± 13.14^a^	28.00 ± 6.45^a^	24.88 ± 11.01^a^	57.25 ± 23.88^b^	31.43 ± 11.07^a^	35.88 ± 10.79^ab^
Osteocytes	8	44.71 ± 5.62^a^	63.71 ± 18.32^ab^	62.13 ± 20.33^ab^	76.25 ± 26.53^b^	50.71 ± 20.31^ab^	60.25 ± 19.14^ab^
Bone marrow adipocytes	8	8.75 ± 5.82	9.29 ± 7.32	8.75 ± 5.82	13.75 ± 9.16	13.13 ± 11.00	5.00 ± 0.00

C: control; TP: tempeh; RS: soy; DG: daidzein and genistein; LA: *Lactobacillus acidophilus*; DGLA: daidzein and genistein and *Lactobacillus acidophilus*. Results of ANOVA analysis followed by Tukey's post hoc honestly significant difference test showing significant differences between groups, denoted by different superscript letters (^a, b^) and presented as mean ± SD.

### 3.6 Correlation between calcium contents in tissues, biochemical parameters in serum, and bone cells

The association between calcium contents in organs, blood parameters in serum, and bone cells is depicted in Table 6. Calcium contents in the kidney showed a significant inverse relationship with triacylglycerol contents and osteoblasts. In addition, pancreatic calcium contents showed a strong inverse relationship with triacylglycerol contents. Meanwhile, glucose and triacylglycerol contents showed a positive relationship with osteoblasts. Moreover, it is important to note that statistical significance (i.e. a low p-value) does not necessarily indicate a strong or clinically significant correlation between variables. In this study, while the p-values for some of the correlations were significant, it is important to consider the strength and direction of the correlation, as well as the context in which it occurs. In some cases, moderate correlations may still have important implications for the research question being investigated.

**Table 6:** Spearman's correlation result between calcium contents, blood parameters, and femoral bone cells.

Correlations		Correlation coefficient	Significance*
Calcium contents in tissues and blood parameters	Kidney – TG	−0.453	0.003
	Pancreas – TG	−0.368	0.018
Calcium contents in tissues and bone cells	Kidney – OTB	−0.404	0.008
Blood parameters and bone cells	GLU – OTB	0.392	0.009
	TG – OTB	0.353	0.022

GLU: glucose; TG: triacylglycerol; and OTB: osteoblast. Correlation analysis between calcium contents in tissues, blood parameters, and femoral bone cells using Spearman's correlation method. Significant correlations (a two-tailed significance test) denoted by * (p < 0.05).

## 4 Discussion

The primary findings of this study were as follows: a modified diet containing daidzein and genistein had significantly high osteoblasts and osteocytes amount, and the presence of *L. acidophilus* significantly increased the calcium concentration in the femurs of healthy rats.

Numerous studies have reported the effects of daidzein on bone cells and metabolism. For instance, daidzein enhances osteoblast development at various stages (from osteoprogenitors to terminally differentiated osteoblasts), and its influence on bone morphogenetic protein (BMP) production in mature osteoblasts is also reported. BMP2 synthesis is markedly increased in response to daidzein, suggesting that some of the effects of daidzein on the cell may be mediated via increased BMP production by osteoblasts [24]. Similarly, daidzein regulates the growth and development of osteoblastic OCT1 cells by stimulating the BMP-2/Smads pathway [25]. BMP-2 operates on bone cells by binding cell surface receptors and triggering the phosphorylation of Smads, which translocates into the nucleus and then activates the transcription of bone-specific genes [[26], [27], [28]]. Furthermore, daidzein promotes bone differentiation, mineralization, and bone formation in both early- and late-stage osteoblasts [29].

Another explanation of these findings may be that daidzein stimulates osteogenesis by facilitating proliferation, differentiation, and antiapoptosis in human osteoblast-like MG-63 cells via the activation of MEK/ERK and PI3K/Akt in an ER-dependent manner [30]. Moreover, isoflavones primarily signal via estrogen receptors, which minimize bone loss by inhibiting osteoclasts [31]. Therefore, the results of the present study are consistent with those of previous studies on the role of isoflavones in enhancing the metabolism of bone cells. Although daidzein and genistein increased osteoblasts and osteocytes, they did not enhance calcium contents in tissues, particularly bone femurs. Bone CYP27B1 gene expression has been shown to be increased in mineralizing osteoblasts and response to a high-calcium diet [32].

In this study, *L. acidophilus* enhanced the calcium content in the femur in healthy rats. Until recently, information about how probiotic microorganisms potentially influence bone density and mineral metabolism has been scarce [33]. Some probiotics have prevented age-related bone loss in senescence-accelerated mice [34], and others have increased bone mineral density in mice [35]. A decrease in bone mineral density in the femur, the spine, and the whole body has been associated with ovariectomy. Previous studies have shown that *L. acidophilus* was more successful at preventing this [36]. The present study showed that the calcium content of the femur was significantly higher in the *L. acidophilus* group compared with the control group. The increased calcium content in the femur and tibia can be attributable to the increased calcium absorption in the distal colon [37]. A clinical study reported that increasing calcium contents in the femur of healthy older women could be attributable to the absence of a dysbiosis effect that could promote bone loss. There is evidence that the gut microbiota regulates bone mass by influencing calcium absorption and the synthesis of serotonin, which interacts with bone cells [38]. The present study's findings suggested that *L. acidophilus* positively enhances the calcium content in bones, but further studies are needed to confirm these results.

Although daidzein and genistein improved osteoblasts and osteocytes and *L. acidophilus* increased calcium contents in the femur, in this study, no evidence for a synergistic effect of isoflavones and probiotics on calcium homeostasis and bone formation could be obtained. Perhaps the major reason for this observation is the usage of healthy rats. The condition of bone disorders can reveal synergistic effects. Probably, ovariectomized animal models mimicking osteoporosis might have shown the advantages of isoflavones and probiotics. Previous studies have found that daidzein reduces the osteoclastogenic effect in ovariectomized mice [39] and that *L. acidophilus* promotes bone heterogeneity and prevents bone loss in osteoporotic mice [14].

In this study, isoflavones and *L. acidophilus* redistributed calcium between tissues in rats. These findings demonstrate that compared with the control group, the daidzein and genistein, *L. acidophilus*, and the combination of daidzein, genistein, and *L. acidophilus* groups showed significantly lower calcium contents in the heart and kidneys (Table 4). However, in these groups, increased calcium contents were observed in the femur. It seems that the combination of daidzein, genistein, and *L. acidophilus* may regulate calcium redistribution in tissues, especially from the heart and kidneys to bones. Interestingly, this phenomenon can be observed in the inverse correlation between calcium contents in tissues and bone cells (Table 6). A significant inverse association was observed between calcium contents in kidneys and the number of osteoblasts (the lower the calcium contents in kidneys, the higher the number of osteoblasts). The findings of this study suggest that the combination of daidzein, genistein, and *L. acidophilus* may affect calcium redistribution, which is related to osteoblasts physiology. Moreover, since biochemical parameters regulate the metabolic control system, calcium redistribution affects bone cells associated with blood morphology, in accordance with the positive association observed between blood parameters and bone cells. Evidence from previous studies shows that a high glucose concentration increases osteoblast expression [40].

A significant change was observed in the effect of soybeans on triacylglycerol concentrations. Soybean intake significantly decreased triacylglycerol concentrations compared with the standard diet. This decrease was triggered by the phytate concentration in soybeans [41]. Furthermore, the phytate content of unfermented soy is higher than that of fermented soy products such as tempeh because phytates are mobilized during the fermentation of legumes by *Rhizopus* during the synthesis of tempeh by the activity of the enzyme phytase, which decreases the concentration of phytic acid [42].

The major contribution of this study is using probiotics and isoflavones together as an intervention to evaluate calcium status in tissues and skeletal architecture of healthy rats. Our findings have important implications for human health as our obtained results suggest that combining isoflavones and probiotics can positively affect bone health by improving bone calcium levels and promoting bone cell proliferation. These findings suggest a potential role for isoflavones and probiotics in improvement of calcium status and in the prevention of osteoporosis, a common and debilitating condition that affects millions of people worldwide. Furthermore, our study provides insight into the potential mechanisms underlying the observed effects of isoflavones and probiotics on bone health, which can aid in the development of more targeted and effective interventions. The results of our research can be the basis for developing nutritional recommendations or dietary supplements for people at high risk of bone diseases due to calcium deficiency.

This study has significant limitations since only selected biochemical and skeletal parameters were analyzed. Moreover, we conducted our study on healthy rats without any bone metabolism disruption. Despite the fact that only healthy female rats were used in this study, we acknowledge the potential limitations of our study concerning gender differences. Our findings provide important insights into the potential benefits of isoflavones and probiotics on bone health. They can serve as a foundation for further investigation in both male and female populations with osteoporosis. Future studies could address the potential gender differences in response to these interventions and further elucidate the underlying mechanisms.

## 5 Conclusions

The combination of daidzein, genistein, and *L. acidophilus* may be potentially beneficial for the calcium content in the bone and bone cells. However, the synergistic effect of isoflavones and probiotics on calcium status and bone health was not observed in healthy rats.

## Ethics approval

The Chairman of the Local Ethics Committee for Experimental Animals has permitted the present study.

## Consent for publication

All authors consent to the publication of the manuscript.

## Funding statement

This work was supported by the statutory research from Poznań University of Life Sciences with No. 506-786-00-03. In addition, this study secured the 2021 Young Scientist Research Grant from Faculty of Food Science and Nutrition, Poznań University of Life Sciences, for I.A.H.

## Author contribution statement

Conceptualisation: I.A.H and J.S.; Data curation: I.A.H.; Formal analysis: I.A.H.; Funding acquisition: I.A.H. and J.S.; Investigation: I.A.H.; Methodology: I.A.H., M.K., M. Sc., E.P·O., M. Sa., and J.S.; Project administration: I.A.H.; Resources: I.A.H.; Software: I.A.H.; Supervision: J.S.; Validation: I.A.H. and J.S.; Visualisation: I.A.H.; Writing–original draft, I.A.H.; Writing–review and editing, I.A.H. and J.S. All authors have read and agreed to the published version of the manuscript.

## Availability of data

All data generated or analyzed during this study are available from the corresponding author on reasonable request.

## Additional information

No additional information is available for this paper.

## Declaration of competing interest

The authors declare that they have no known competing financial interests or personal relationships that could have appeared to influence the work reported in this paper.
