# Corrigendum to “Isoflavones and probiotics effect on bone calcium and bone cells in rats” [Heliyon 9(6) (June 2023) e16801]

**DOI:** 10.1016/j.heliyon.2023.e19589

**Published:** 2023-09-04

**Authors:** Iskandar Azmy Harahap, Maciej Kuligowski, Marcin Schmidt, Paweł Kurzawa, Ewa Pruszyńska-Oszmałek, Maciej Sassek, Joanna Suliburska

**Affiliations:** a Department of Human Nutrition and Dietetics, Faculty of Food Science and Nutrition, Poznań University of Life Sciences, Poznań, Poland; b Department of Food Technology of Plant Origin, Faculty of Food Science and Nutrition, Poznań University of Life Sciences, Poznań, Poland; c Department of Biotechnology and Food Microbiology, Faculty of Food Science and Nutrition, Poznań University of Life Sciences, Poznań, Poland; d Department of Clinical Pathology, Poznań University of Medical Sciences, Poznań, Poland; e Department of Animal Physiology, Biochemistry and Biostructure, Faculty of Veterinary Medicine and Animal Science, Poznań University of Life Sciences, Poznań, Poland

## Introduction

In the original published version of this article, the typesetters have used the outdated version, the authors reviewed the proof and approved it before publication. An outdated version of the paper was published. We will publish a final version that the authors and editors approved. The correct version of the manuscript will replace the outdated version. The authors apologize for the errors. Both the HTML and PDF versions of the article have been updated to correct the errors.

## Declaration of competing interest

The authors declare no conflict of interest.

